# Dynamic Evolution Mechanism of Digital Entrepreneurship Ecosystem Based on Text Sentiment Computing Analysis

**DOI:** 10.3389/fpsyg.2021.725168

**Published:** 2021-09-20

**Authors:** Jiahui Li, Meifang Yao

**Affiliations:** School of Management, Jilin University, Changchun, China

**Keywords:** text sentiment analysis, deep learning, digital entrepreneurship ecosystem, dynamic evolution, computing analysis

## Abstract

To solve the limitations of the current entrepreneurial ecosystem, the research on the digital entrepreneurial ecosystem is more meaningful. This article aims to study the dynamic evolution mechanism of the digital entrepreneurship ecosystem based on text sentiment computing analysis. It proposes an improved Bi-directional long short-term memory (Bi-LSTM) model, which uses a multilayer neural network to deal with classification problems. It has a higher accuracy rate, recall rate, and F1 value than the traditional LSTM model and can better perform sentiment analysis on text. The algorithm uses the optimized Naive Bayes algorithm, which is based on Euclidean distance weighting and can assign different weights to the final classification results according to different attributes. Compared with the general Bayes algorithm, it improves the calculation efficiency and can better match the digital entrepreneurial ecosystem, which is evolving dynamically, predicting and analyzing its future development. The experimental results in this article show that the improved Bi-LSTM is better than the traditional Bi-LSTM model in terms of accuracy and F1 value. The accuracy rate is increased by 1.1%, the F1 value is increased by 0.6%, and the recall rate is only <0.2%. Running on the Spark platform, although 3% accuracy is sacrificed, the running time is increased by 320%. Compared with the traditional cellular neural network (CNN) algorithm, the accuracy rate is increased by 4%, the recall rate is increased by 14%, and the F1 value is increased by 9%, which proves that it has a strong non-linear fitting ability. The performance improvement brought by the huge data set is very huge, which fully proves the feasibility of the digital entrepreneurship ecosystem.

## Introduction

Since 2015, supply-side reforms have come into being, emphasizing that innovation should be the first engine driving economic development, stimulating innovation potential and optimizing industrial structure. Entrepreneurship activities are very important for improving regional economic development, talent gathering, and promoting the sustainable development of industries. However, the technological content of entrepreneurship of Chinais still low, and the strong scientific and technological strengths have not been absorbed by entrepreneurial enterprises. Under different technological systems and market environments, issues such as the allocation of scientific and technological resources and the efficiency of resource utilization have a significant impact on the sustainable development of enterprises. The entrepreneurial practice of Chinese enterprises shows that the “national system” type of entrepreneurial support has not made the national entrepreneurial system out of the “island phenomenon” in technological innovation. The gestation and growth processes of entrepreneurship are nested in a series of complex systems that influence each other. In these systems, each subsystem interacts with other systems and individuals, affecting all aspects of entrepreneurial activities. In recent years, the construction of an entrepreneurial ecosystem has become an inevitable way to cultivate unique regional competitive advantages. The self-organization and self-regulation capabilities of the entrepreneurial ecosystem play an important role in the sustainable development of their internal enterprises. Due to the lack of a scientific evaluation mechanism, the construction of entrepreneurial ecosystem of China is still in the exploratory stage, and the promotion of typical cases established by following the trend can easily lead to the waste of resources, such as repeated construction. “No Measurement, No Improvement,” how to use a reasonable evaluation system to provide targeted and operable suggestions for the optimization of the entrepreneurial ecosystem is worthy of further research. As a diversified, mutually beneficial, and win-win innovation and entrepreneurship system, the business ecosystem is of great significance to the formulation of innovation and entrepreneurship policies by analyzing the impact of different factors on its operation.

The entrepreneurial ecosystem is composed of three aspects: entrepreneurial enterprises, stakeholders, and the entrepreneurial environment. The entrepreneurial entities are connected by formal or informal relationships. Different communities influence and depend on each other, and evolve together over time, and they have obvious regional characteristics. On the one hand, the existence of the entrepreneurial ecosystem can save excessive transaction costs and organization costs. On the other hand, it can provide a more advantageous development environment for new start-ups in an unstable market environment, and improve the competitiveness of start-ups, which is a guarantee keyway to the quality and vitality of new ventures. However, the effective operation of the entrepreneurial ecosystem is inseparable from the coevolution and mutual synergy between the constituent communities and elements. Therefore, through efficiency evaluation and analysis of influencing factors, the various elements within the entrepreneurial ecosystem, the linkages between the elements, and the driving force of the linkages are discussed. It has an important guiding role in the formation of a real-time, creative, and orderly entrepreneurial ecosystem.

At present, the Internet is developing rapidly, and many people are paying attention to the digital entrepreneurial ecosystem. The following people have unique insights on this. Singh SK proposed a new framework for a spell-check system that extracts user reactions, emotions, and opinions from social media text (SMT). User opinions are extracted from their written texts on social media and based on SMT sentiment scores, using dictionary-based methods and binary classifiers, which are classified as positive or negative opinions. The dictionary-based method uses the opinion verb dictionary (OVD) to extract the sentiment of opinion verbs that appear in SMT. This OVD contains only opinion verbs and their sentiment scores. The various steps of the framework have been discussed, such as lowercase conversion, tokenization, spell-checking, part-of-speech tagging, stop word elimination, stemming, sentiment score calculation, and SMT classification. Although Singh introduced the new concept of threshold negative parameters to facilitate the extraction of text information data, the efficiency of this extraction method is slightly low. If the optimized Bayes algorithm is used, the efficiency can be greatly improved (Singh and Sachan, 2019). Le X proposed that the rapid popularization of digital technology with new functions has profoundly changed the competitive environment and reshaped traditional business strategies and processes. Such technologies have also spawned new ways of collaboration, using resources, service design, development, and deployment based on open standards and shared technologies. At the micro-level, digital technology has also reshaped the mindset of entrepreneurs, thereby affecting their decision-making process. Digital entrepreneurship includes venture capital and the transformation of existing enterprises through the creation of new digital technologies and new uses of these technologies. At present, many countries regard digital entrepreneurship as an important pillar for the development of the digital economy, and it is necessary to have a detailed understanding of digital entrepreneurship. Although Le has formulated the relevant content of digital entrepreneurship, he has not made an in-depth dynamic evolution of the digital entrepreneurship system, which makes it impossible for him to estimate the future development of digital entrepreneurship (Le et al., 2019). Allenby CE discovered that digital health entrepreneurship is a topic widely discussed in non-professional and professional media. Innovators in this field continue to raise large amounts of funds to develop applications and devices that will change the way health care is managed in the United States. As many as one-third of American consumers use wearable devices and mobile health applications, which provide important opportunities for putting health care in the hands of consumers. However, it remains to be seen who will play the greatest role in this field. Technology giants, biopharmaceutical companies, and start-ups all have the opportunity to take advantage of new areas and change the way health care interacts with patients. Although entrepreneurs are in a leading position in the technology industry, they lack the regulatory and healthcare expertise of large pharmaceutical companies. However, as start-ups and large technology companies continue to promote innovation in this field, the pharmaceutical industry will not be able to continue to pack lightly. Companies that decide to take stock in this market will have to decide on how to interact with the health care sector. He discovered that digital entrepreneurship has great potential for development. He focused his attention on the development of medical and health care, but this development relied on large local technology companies and biopharmaceutical companies. He did not take advantage of the convenience of text sentiment calculation and analysis. If based on text sentiment calculation analysis to dynamically evolve digital entrepreneurship, then he can develop on his own (Allenby et al., 2018).

The innovations of this article are the following: (1) Using text sentiment calculation and analysis methods, combined with deep learning algorithms, the traditional Bayesian algorithm is improved and optimized, and the efficiency is improved. (2) Putting forward the theory of the digital entrepreneurship ecosystem, discovering the huge potential for future development, and analyzing the important components of the system and its influencing factors. (3) Comparing the algorithm with other models, a better Bi-LSTM model is obtained. It is found that the running time under the Spark platform is much shorter than the running time on the native model.

The first section of this article introduces the background and significance of the current domestic and foreign entrepreneurial ecosystems, and cites several references that have important results in emotional computing, and also describes the innovations of this article. The second section briefly introduces the creation of ecological theory, core competitiveness theory, text sentiment calculation analysis, naive Bayes algorithm, and entrepreneurial process theory. In the introduction of naive Bayes algorithm, it proposes its improvement and optimization. In the later Bayesian algorithm theory. The third section introduces the experimental purpose, experimental procedures, and experimental methods of this article. The fourth section focuses on the analysis of the influencing factors of sentiment computing in the dynamic evolution of the digital entrepreneurship ecosystem, compares the performance of various traditional algorithms, and then analyzes and introduces the optimized and improved text sentiment computing algorithms in this article. The fifth section is a summary of this article and puts forward the shortcomings in the article and the outlook for the future.

## Dynamic Evolution Mechanism of Digital Entrepreneurship Ecosystem Based on Text Sentiment Calculation Analysis

### Entrepreneurship Ecosystem

The concept of an entrepreneurial ecosystem originates from the combination of entrepreneurial theory and ecological theory. To be precise, it is a reexamination of entrepreneurial theory from an ecological perspective. With the advancement of science and technology and the shortening of product life cycles, the knowledge required for product innovation is distributed in fragments, and the process of technological innovation shows more non-linear characteristics. The effect of research and development (R&D) investment on R&D performance also depends on the collaboration of various entrepreneurial entities. From the beginning of the establishment to the final demise, start-ups have always been in dynamic communication with the environment in which they are located (the inflow of key elements and product output). The competition and cooperation between different entrepreneurs are complicated, but they have always maintained the entire entrepreneurial system. These characteristics make the entrepreneurial process naturally have an analysis function of ecological significance (Shen et al., 2018). Moore (1996) first proposed the concept of the business ecosystem and analyzed the relationship between entrepreneurial enterprises and their related multiple entrepreneurial entities from an ecological perspective, setting a precedent for entrepreneurial ecological research. After that, many scholars have conducted more in-depth research on entrepreneurial ecology (Gabison, 2019).

The ecosystem needs the inflow and outflow of material and energy to maintain the normal operation of the system. For business ecology, this means the need to study the creation and development of new businesses in various fields related to the business environment and business resources. Gnyawali and Fogel (1994) divided the entrepreneurial environment into five dimensions: financial support, non-financial environment, policy regulations, socioeconomic support, and entrepreneurship and management skills, and the impact of these factors on entrepreneurial performance in the entrepreneurial ecosystem perform analysis (Chen, 2021). Li et al. (2011) has very in-depth research in the field of entrepreneurship. He has conducted multidimensional analysis on the relationship between entrepreneurial networks and entrepreneurial performance. In the entrepreneurial environment, entrepreneurial resources have a positive impact on the growth and performance of new ventures. Verify shows (Hutson, 2018). Only when organisms exchange material and energy with the environment can they ensure their development, and entrepreneurial companies can only ensure their sustainable development if they fully utilize and integrate resources and the environment. The entrepreneurial ecosystem has the characteristics of regionality, diversity, symbiosis, external spillover, self-organization, and self-regulation. Many literature studies have conducted relevant research on its regionality and diversity, but the research on its self-organization and self-regulation is still relatively scarce. At present, with the further development of the entrepreneurial ecological theory, the research on the entrepreneurial ecosystem has gradually shifted from its conceptual framework to the analysis of its dynamic evolution. The method of metaphor alone cannot fully play the role of ecology in the entrepreneurial field. Think about the integration of the two from a deeper level (Nuseir, 2018).

### Core Competitiveness Theory

The core competitiveness theory focuses on the ability to integrate related technologies and knowledge, and at the same time points out that the core competence is transferable between organizations and can be transferred to all members of the enterprise in accordance with the organizational structure (Ishmukhametov et al., 2020). Domestic experts and scholars have carried out rich and in-depth research on this theory. The earliest domestic research on core competitiveness was done by Professor Jin Bei of the Chinese Academy of Social Sciences, which can make enterprises stand out from the competition (Miao and Chonghui, 2018). The core competitiveness has the following characteristics: user value, user evaluation, and feedback are the most objective and direct standards to measure the competitiveness of an enterprise. The core competitiveness can be improved only by continuously improving the quality of products and services and by increasing user satisfaction. Occupancy, the company has a long-term occupation of certain necessary development resources, which makes itself have a unique advantage different from other competitors. This resource is difficult to obtain by other companies and will be occupied by the company for a long time; malleability, the core competitiveness of the company can be extended from ideas or technologies to products and services and maintained market advantages. Uniqueness, core competence can give enterprises an advantage in a series of products or services. There are differences in the core competitiveness of different enterprises. This kind of competitiveness is gradually formed in the long-term development of enterprises and has a high degree of imitation (Liu et al., 2018).

### Text Sentiment Calculation Analysis

**Figure 1:**
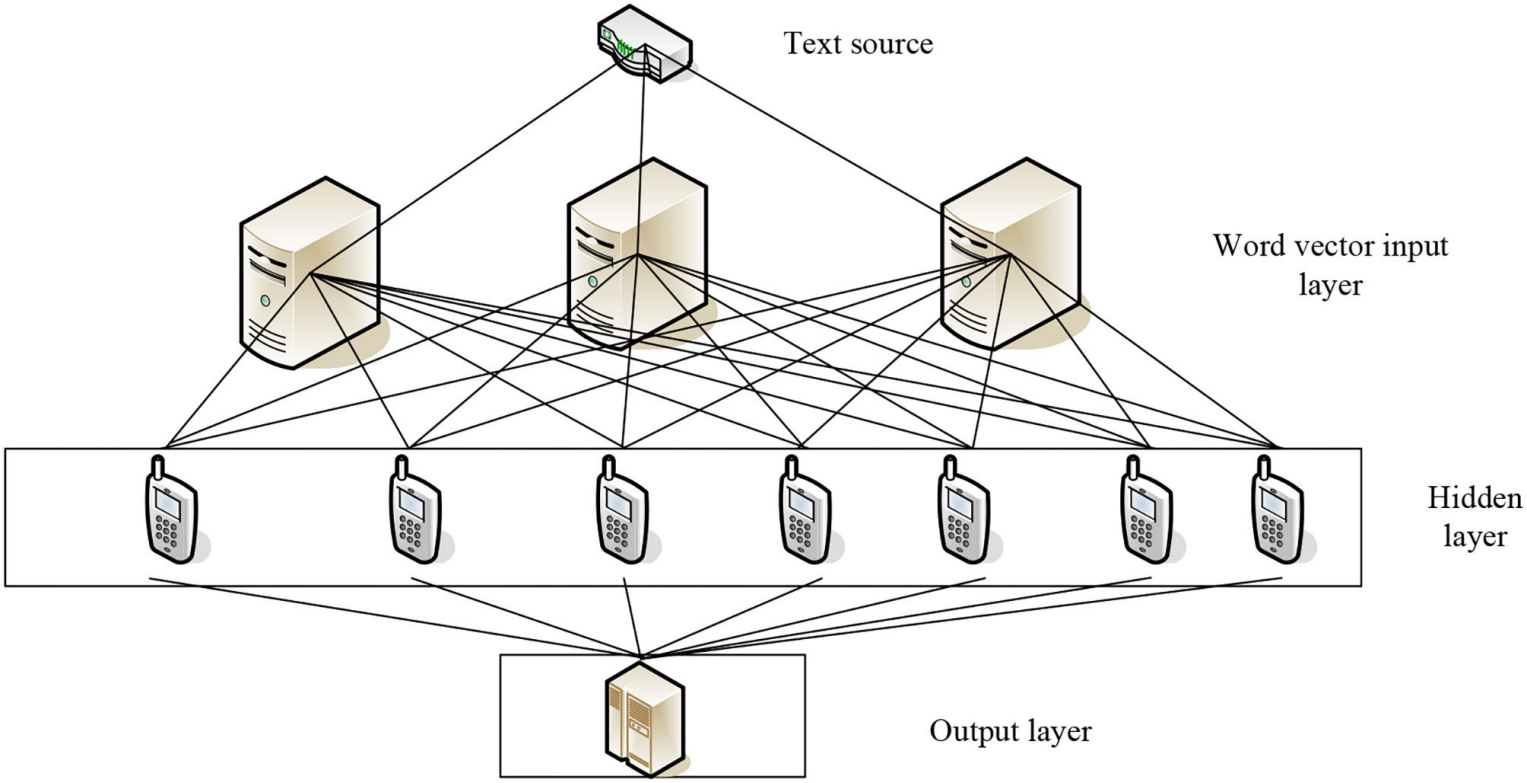
LSTM model.

As shown in Figure 1, the text sentiment calculation analysis model is built on the LSTM artificial neural network. The word vector is extracted from the top text source, and the input layer is the input word vector for sentiment calculation analysis, like the hidden layer input data, training, and filtering. After that, select the data that suit the requirements for output (Frank, 2018). In recent years, sentiment analysis has flourished in computer science, and a large number of articles on sentiment analysis of text have also appeared in major well-known natural language processing-related conferences. According to the granularity of the text, sentiment analysis can be divided into word-level, sentence-level, and text-level. The first two are fine-grained, and the latter are coarse-grained (Zeng et al., 2021). Because an article often has multiple sentences expressing different emotions, and words are the smallest unit of expressing sentence emotions, which are likely to be affected by other vocabulary or sentence patterns, most research are more inclined to use sentences as the granularity. Taking into account complicated situations such as the omission of evaluation objects in emotional expression, experiments have proved that this work can provide a certain basis for emotional analysis based on supervised learning. In terms of sentiment classification, methods based on rules and machine learning are more common. Among unsupervised classification methods, sentiment dictionaries and their construction work also play an important role. A sentiment dictionary is constructed by integrating three parts of psychology domain vocabulary, WordNet ontology dictionary, and commonly used slang words on the Internet. Then, based on this dictionary, it analyzes the incentives and laws of emotions of people in three different cities over time (Chang and Huo, 2018). It can also combine information increment, support vector machine, decision tree, and naive Bayes algorithm for feature selection and sentiment classification, and use positive and negative feature mean to evaluate sentiment similarity; construct a method based on graph learning and semi-supervision, a classifier of Weibo sentiment polarity is constructed. This classifier takes into account the community relationship of Weibo users and the similarity of Weibo text. Experiments have shown that this classifier can replace part of the manual annotation work (Hamdi et al., 2018). After finishing the work of removing stop words and feature extraction on Twitter text, the improved positive feedback neural network method is used to classify the sentiment tendency of the text. Although satisfactory results are finally achieved, the algorithm is trained when the text is too large. The usefulness of the memory needs to be optimized; it can be found that long text and short text have a high degree of overlap in terms. Therefore, based on the characteristics of short text and traditional feature filtering methods, the long text features and short text common features are left, and the superior information is added to the short text classification process (Nam and Lee, 2019). An improved conditional random field model is also proposed, which treats the tendency analysis of phrase sentiment as sequence labeling (Hahn et al., 2020). A method for calculating the similarity of comments posted by users based on cosine similarity is proposed, and each text is assigned a value of emotional orientation strength from −2 (negative) to +2 (positive). The result is similar to the traditional shell, where maximum entropy and support vector machines are all improved (Li et al., 2020); linguistic knowledge is integrated into the traditional sentiment classification, a large number of rules are expanded, and the improved N-grams model is used to solve the language morphology where there are more complicated sentence patterns in characteristics (Kotelnikov, 2018). However, the purely rule-based method requires domain experts to define a large number of rules, which is costly and has limitations. In recent research results, concept-level sentiment analysis is often be mentioned; a concept-level sentiment analysis system is constructed, which integrates semantic analysis, subjectivity detection, irony syntax detection, topic positioning, and other multidimensional, experimental. It is proved that this method can solve the performance defects of some supervised sentiment analysis algorithms in different situations (Singh and Sachan, 2021); through the use of the conceptual hierarchical model to extract text emotional features, extract the semantics of the text, and finally explore some microblog text and natural semantic relevance of language text; it proves that the performance of sentiment analysis at the concept level is better than the performance of sentiment analysis at the lexical level. In addition, in the sentiment analysis corpus, the corpus that does not contain sentimental tendencies is also of practical value. There is a map-reduce-based e-commerce user opinion recognition framework, and new dictionary-based technology is used to mine the products. Neutral evaluation to correct the results of these opinions being classified as positive or negative, to reduce the error of sentiment analysis (Bhagat and Mane, 2019; Schmidt et al., 2020).

### Naive Bayes Algorithm

Conditional probability refers to the occurrence of event B as the premise. In this case, the probability of occurrence of event A is recorded as P(A|B).

Let A and B be two events in the sample space Ω, if P(B) > 0, then it is called as shown in Equation 1:


(1) $$\begin{array}{l} P\left(A|B\right)=\frac{P\left(AB\right)}{P\left(B\right)} \end{array}$$

Event B occurs first. On this basis, the probability that event A will occur. Among them, the probability P(B) of event B is called the prior probability, P(A|B) is the probability that event A will occur based on the occurrence of event B, this probability is called the posterior probability of event A, P (AB) represents the probability that both events A and B will occur. Let A and B be events in the sample space Ω, if P(B) > 0, then equation 2 is described as follows:


(2) $$\begin{array}{l} P\left(AB\right)=P\left(B\right)P\left(A|B\right) \end{array}$$

If *P*(*A*_1_*A*_2_*A*_3_…*A*_*n*−1_) > 0, then


(3) $$\begin{array}{l} P\left(A_{1}A_{2}A_{3}...A_{n}\right)=P\left(A_{1}\right)P\left(A_{2}|A_{1}\right)...P\left(A_{\text{n}}|A_{1}A_{n}\right) \end{array}$$

Assuming that B_1_, B_2_, …B_n_ is a self of the sample space Ω, then B_1_, B_2_, …B_n_ will be mutually exclusive, and $\overset{n}{\underset{j=1}{⋃}}B_{j}=\Omega$, if P(B) > 0, then any event A has


(4) $$\begin{array}{l} \text{P(A)}=\overset{n}{\underset{\text{j}=\text{1}}{\sum }}P\left(B_{j}\right)P\left(A|B_{j}\right) \end{array}$$

From the above, we can integrate the two formulas into one formula, which is the formula of Bayes' theorem:


(5) $$\begin{array}{l} P\left(B_{j}|A\right)=\frac{P\left(AB_{j}\right)}{P\left(A\right)}=\frac{P\left(B_{j}\right)P\left(A|B_{j}\right)}{\overset{n}{\underset{j=1}{\sum }}P\left(B_{j}\right)P\left(A|B_{j}\right)} \end{array}$$

The most likely hypothesis F_*j*_ ∈ *F* for a given sample C is selected from a set of candidate hypotheses f. This hypothesis or category is called the maximal posterior hypothesis, namely


(6) $$\begin{array}{l} f_{MAP}=\underset{f_{j}\in F}{\arg}\max P\left(f|C\right) \end{array}$$

(7) $$\begin{array}{l} =\underset{f_{j}\in F}{\arg}\max\frac{P\left(C|f\right)P\left(f\right)}{P\left(C\right)} \end{array}$$

(8) $$\begin{array}{l} =\underset{f_{j}\in F}{\arg}\max P\left(C|f\right)P\left(f\right) \end{array}$$

Under certain circumstances, assuming that the prior probability of each hypothesis in F is the same, just consider P(C|f) to find the most probable hypothesis, so P(C|f) is called when f The likelihood of data C, where the hypothesis that maximizes P(C|f) is called the maximum likelihood hypothesis (Lutfullaeva et al., 2018):


(9) $$\begin{array}{l} f_{ML}=\underset{f_{j}\in F}{\arg}\max P\left(C|f\right) \end{array}$$

It can be seen from the Bayesian formula that the posterior probability P(f|C) depends on the product of P(C|f) P(f), which is also the most important idea of the Bayesian classification algorithm. According to Bayes' theorem:


(10) $$\begin{array}{l} P\left(d_{i}|y_{1},y_{2},...y_{\text{n}}\right)=\frac{P\left(y_{1},y_{2},...y_{\text{n}}|d_{i}\right)P\left(d_{i}\right)}{P\left(y_{1},y_{2},...y_{\text{n}}\right)} \end{array}$$

Among them, P_*i*_ is the probability that the text belongs to the category *d*_*i*_ in the training text. In the partially selected categories, Bayesian classification assumes that the attributes in the classification are independent of each other, then Equation 11 is given as follows:


(11) $$\begin{array}{l} P\left(y_{1},y_{2},...y_{\text{n}}|d_{i}\right)=\overset{n}{\underset{k=1}{\prod }}P\left(y_{k}|d_{i}\right) \end{array}$$

Substituting the prior probability *P*(*y*_1_, *d*_*i*_), *P*(*y*_2_, *d*_*i*_), …*P*(*y*_*n*_, *d*_*i*_), calculated from the training set into the above formula, we can get the following Equation 12:


(12) $$\begin{array}{l} P\left(d_{i}|Y\right)=\frac{\overset{n}{\underset{K=1}{\prod }}P\left(y_{k}|d_{i}\right)P\left(d_{i}\right)}{P\left(y_{1},y_{2},...y_{n}\right)} \end{array}$$

The denominator in the formula is a constant, and the Naive Bayes model formula is a maximum value of the calculation formula:


(13) $$\begin{array}{l} {\text{d}}_{NB}\left(Y\right)=\underset{f_{j}\in F}{\arg}\max P\left(d_{i}\right)\overset{n}{\underset{K=1}{\prod }}P\left(y_{k}|d_{i}\right) \end{array}$$

In the above formula, because it is necessary to calculate a large number of product operations, which brings difficulties to the calculation, the formula is modified as follows:


(14) $$\begin{array}{l} {\text{d}}_{NB}\left(Y\right)=\underset{f_{j}\in F}{\arg}\max\left[\log P\left(d_{i}\right)+\overset{n}{\underset{K=1}{\sum }}P\left(y_{k}|d_{i}\right)\right] \end{array}$$

The naive Bayes classification algorithm simplifies the estimation of the prior probability when the assumption of independence of distribution is established, without considering the correlation between different attributes. The process of predicting the sample to be predicted is simple, the classification complexity is low, the speed is fast, and the cost in the calculation process is relatively low.


(15) $$\begin{array}{l} P\left(D_{j}|Y\right)=P\left(D_{j}\right)\prod P^{w_{k}}\left(y_{k}|D_{j}\right) \end{array}$$

Among them, the weight of the attribute A is w_*k*_, that is, the weight of the P(Y|D_j_). The larger w_*k*_, the greater the importance of the attribute to the classification result, which affects the classification result.

### Entrepreneurship Process Theory

Many scholars have conducted in-depth research on entrepreneurial-related theories, such as entrepreneurial characteristics theory, entrepreneurial process theory, entrepreneurial cycle theory, entrepreneurial network theory, etc., discussing the factors of entrepreneurial success and failure from multiple aspects. The first to emerge is the entrepreneurial characteristics theory, which believes that the personal characteristics of an entrepreneur (enterprise willingness, risk tolerance, ability to formulate strategies, etc.) play a decisive role in the success of entrepreneurship. With the deepening of entrepreneurial research, scholars have begun to pay attention to the influence of more traits of non-entrepreneurs on entrepreneurial activities, and the research on the entrepreneurial process has gradually attracted the attention of relevant scholars.

Gartner (1985) first proposed that the success of entrepreneurship is not only related to the characteristics of entrepreneurs. The entrepreneurial process is a dynamic, complex, and diverse process. It is a synthesis of many factors in the entrepreneurial process. He compared these related to entrepreneurship. The elements are summarized into four aspects, namely entrepreneurs, stakeholders, entrepreneurial environment, and entrepreneurial practices. Morris et al. (1994) combined previous entrepreneurial research results, regarded the entrepreneurial process as a dynamic process of resource input-product output, and constructed an input-output integration model based on the entrepreneurial process. Xuebing and Gang (2009) believe that entrepreneurship is a process by which entrepreneurs transform new products and new patents into economic benefits by integrating entrepreneurial resources and identifying market opportunities in technological innovation. Entrepreneurship process theory is a very important theory in the field of entrepreneurship. It makes entrepreneurial activity no longer a “black box.” People can analyze the different stages of the entrepreneurial process and explore the impact of different stages on entrepreneurial performance, making the part and the whole static Take into account the dynamics (Lian et al., 2020; Sodhar et al., 2020).

## Experiment on the Dynamic Evolution Mechanism of Digital Entrepreneurship Ecosystem Based on Text Sentiment Calculation Analysis

The key to understanding the digital entrepreneurship ecosystem is to clearly define the core role of the system, which is also the prerequisite for the construction of the system structure model. By combining and analyzing the existing literature, it is found that there is good consistency in the division of the roles of the digital entrepreneurship ecosystem in the existing research, which has laid a good foundation for the following experimental parts.

### Subjects

The digital entrepreneurship ecosystem is a big data ecosystem based on text sentiment calculation analysis. The digital entrepreneurship ecosystem can be divided from a micro and macro perspective. From a micro perspective, the core roles in the big data ecosystem are divided into users and providers and data. From a macro perspective, the system can be divided into a core layer, an extension layer, and an environment layer. The microscopic digital entrepreneurship system is the main research object of this experiment.

**Figure 2:**
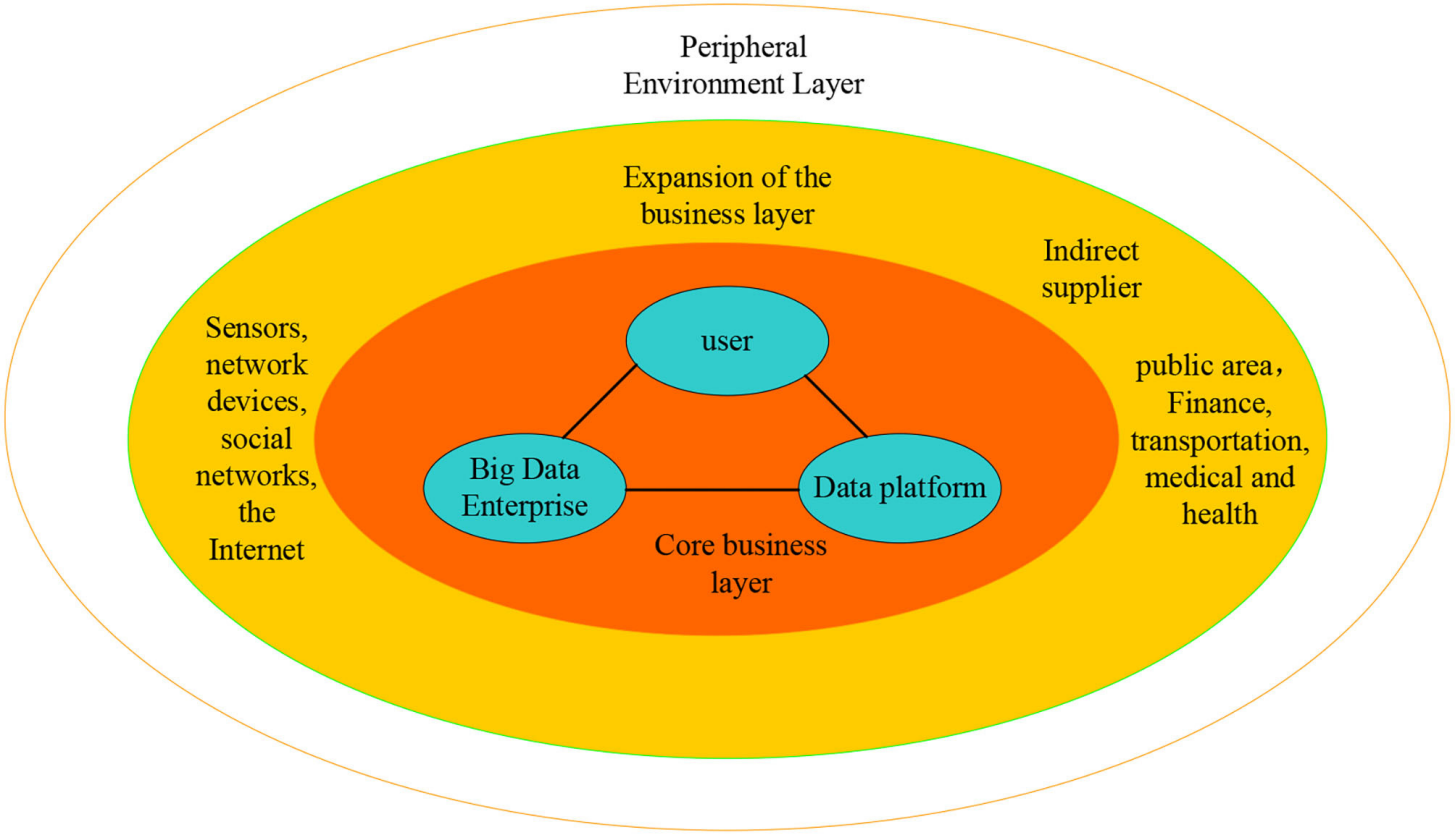
Digital entrepreneurship ecosystem structure model.

As shown in Figure 2, the digital entrepreneurship ecosystem is divided into three levels, namely the core entrepreneurial layer, the extended entrepreneurial layer, and the peripheral environment layer. The core entrepreneurial layer is composed of big data companies, data platforms, and users. Big data companies can be divided into big data core companies and big data niche companies based on the entrepreneurial ecological perspective.

### Purpose of the Experiment

The purpose of the experiment in this article is to analyze the elements of the digital entrepreneurship ecosystem model and then analyze the pros and cons of the algorithm by comparing text-based sentiment computing with other algorithms.

### Experimental Procedure

**Figure 3:**
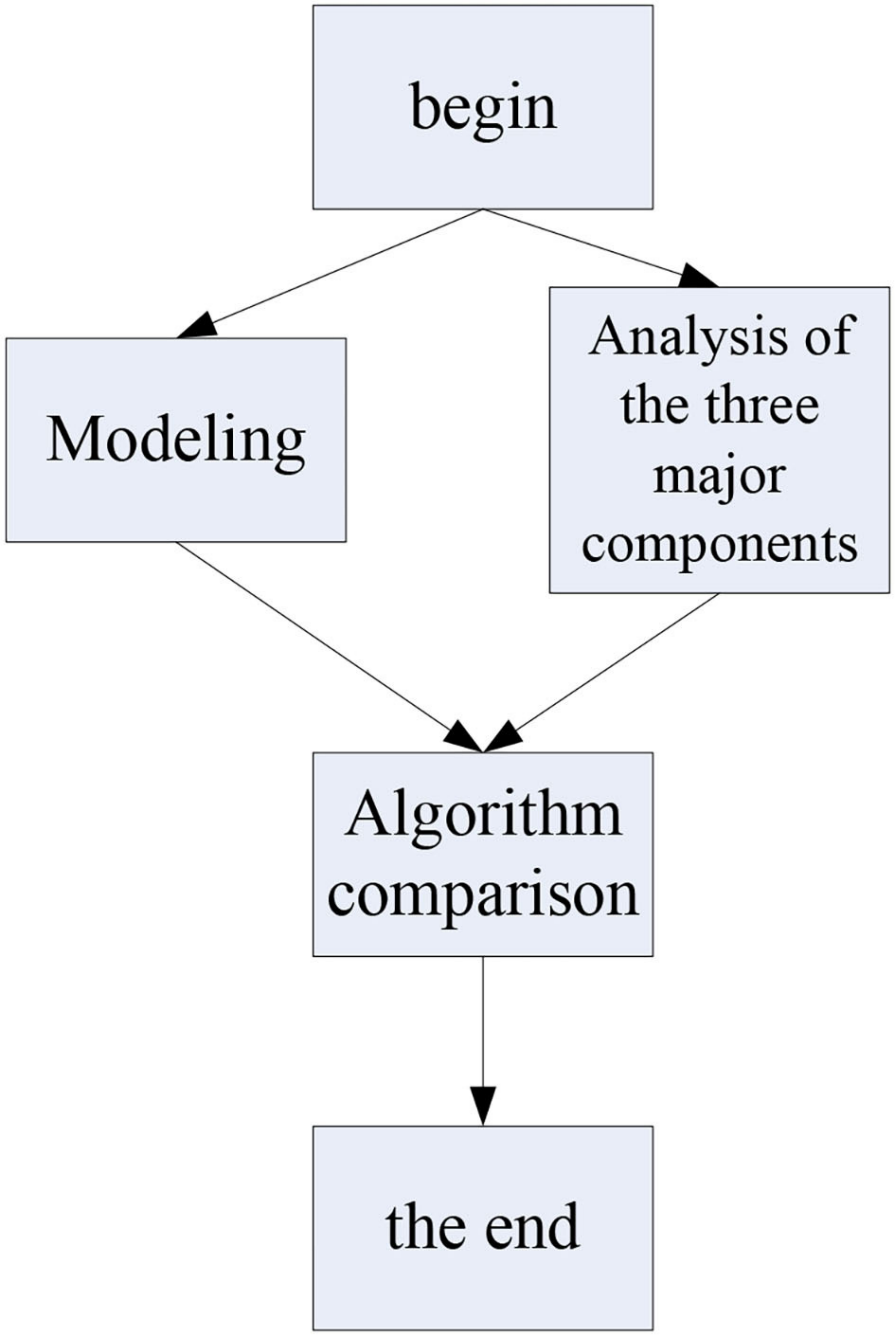
Experimental flowchart.

As shown in Figure 3, the first step of this experiment is to build a digital entrepreneurship ecosystem model, then analyze the construction elements of the digital entrepreneurship model, analyze the key points of its elements and the difference between the manufacturing ecological models, and then compare the algorithms. The cross-experiment method will perform performance screening based on text sentiment computing and other artificial intelligence algorithms, and finally, the experiment ends.

## Dynamic Evolution Mechanism of Digital Entrepreneurship Ecosystem Based on Text Sentiment Computing Analysis

### Influencing Factors

**Figure 4:**
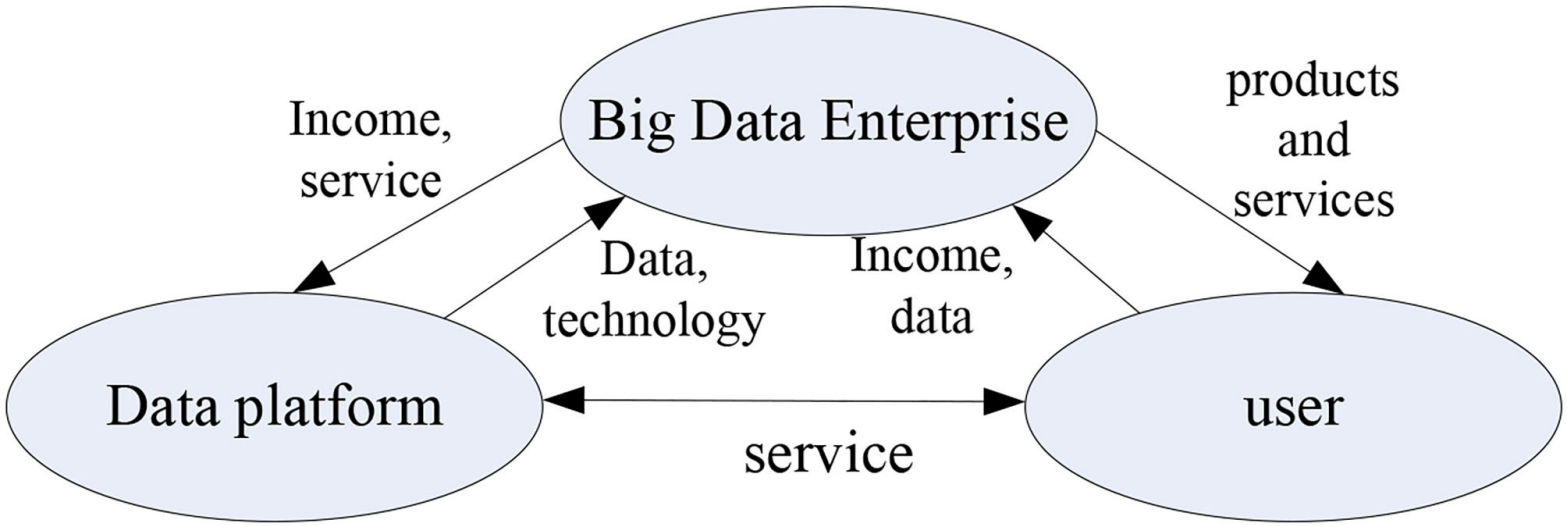
The relationship between the three major elements of the ecosystem.

The relationship between the three core elements of the digital entrepreneurship ecosystem is shown in Figure 4. The relationship between the elements can be understood from two levels. One is from the macro-level of system composition. The second is from the internal components of each level. Among them, big data companies are the main force driving the formation of the digital entrepreneurial ecosystem, which provides users with a wealth of data products and services; the data platform, as an exchange and circulation platform for data resources and a related technical service platform, continues to provide the development of the big data industry. “Raw materials” and technology and service support; users are consumers of data products and services, and the ultimate channel for product value to be realized. Their huge demand for data products and services is the driving force behind the development of the big data industry.

As shown in Table 1, one is the efficient operation of the entrepreneurial ecosystem (M1, M2) brought about by good regional economic conditions, and the other is the operation of the entrepreneurial ecosystem due to the interaction between the maturity of the technology market and the entrepreneurial support institutions. The high efficiency obtained (M3) is the high efficiency brought about by the combined effect of the degree of cooperation between industry, university, and research institute and the characteristics of entrepreneurs (M4). It can be seen that the progress of entrepreneurial activities is closely related to the entrepreneurial environment in which they are located. A relaxed entrepreneurial atmosphere and an active market environment are the keys to the smooth progress of entrepreneurial activities.

**Table 1:** Combination analysis of sufficient conditions for efficient operation of an entrepreneurial ecosystem.

**Variable Name**	**M1**	**M2**	**M3**	**M4**
Regional economic development	•	•	◦	◦
Technology market maturity	◦	◦	•	◦
Government support	◦	◦	◦	◦
Industry-university-research cooperation degree	◦	◦	◦	•
Entrepreneur traits		◦		•
Entrepreneurship support organization scale	◦		•	◦
Raw coverage	0.423	0.415	0.431	0.122
Consistency	0.815	0.785	0.776	0.910

“*•” means that the condition exists, “◦” means that the condition does not exist, and blank means that the condition may or may not exist*.

### Performance Comparison of Algorithm Classification Results

**Figure 5:**
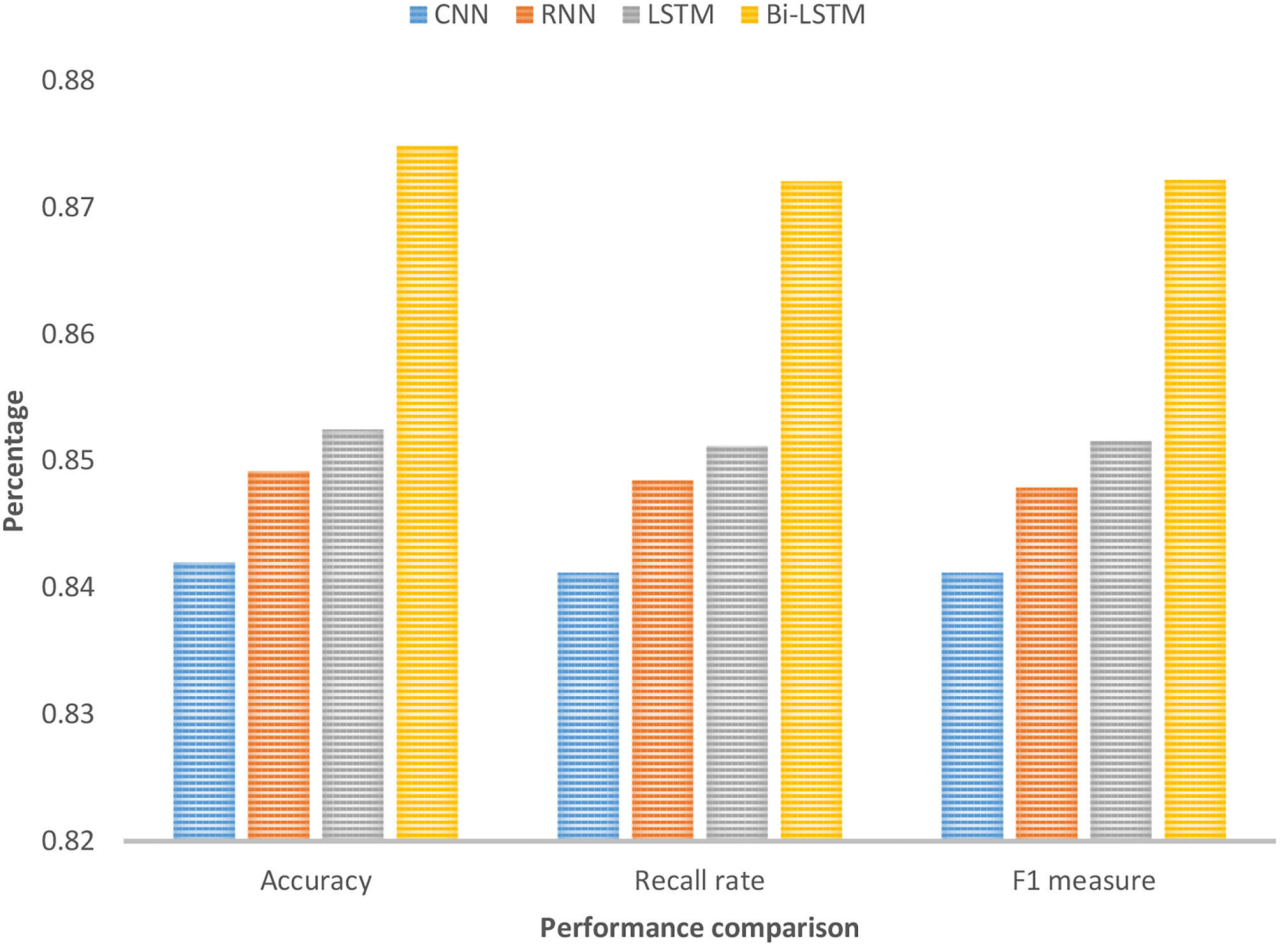
Performance comparison of classification results.

As shown in Figure 5, the overall effect of the Bi-LSTM algorithm is better than other comparative experimental methods, indicating that more effective semantic information can be obtained after considering the contextual information of the input data to improve the accuracy of the results. Bi-LSTM uses a randomly initialized vector to represent the input text data, and then uses a two-way long and short-term memory network structure for text classification. The network selected for the feature expression process is Bi-LSTM, and the number of hidden layer nodes is set to 256. The learning rate is set to 0.01, and the stochastic gradient descent algorithm is used to update and iterate the weight to obtain a stable model. Bi-LSTM is one level higher than the traditional artificial intelligence algorithm in terms of accuracy, recall, or F1 measurement.

As shown in Table 2, the improved Bi-LSTM is superior to other models in terms of accuracy and F1 value, and the recall rate is only lower than the highest 0.2%, which fully proves that this model is reasonable. In addition, it can be seen that the accuracy of the traditional artificial intelligence CNN algorithm is significantly higher than that of LSTM, but the recall rate is lower, which proves that strong non-linear fitting ability of LSTM guarantees its high recall rate.

**Table 2:** Comparative data of different models.

**Model**	**Accuracy**	**Recall Rate**	**F1 Value**
CNN	60.2%	49.7%	54.3%
LSTM	57.4%	63.1%	59.6%
Bi-LSTM	63.2%	64.0%	63.4%
Improved Bi-LSTM	64.2%	63.7%	63.8%

**Figure 6:**
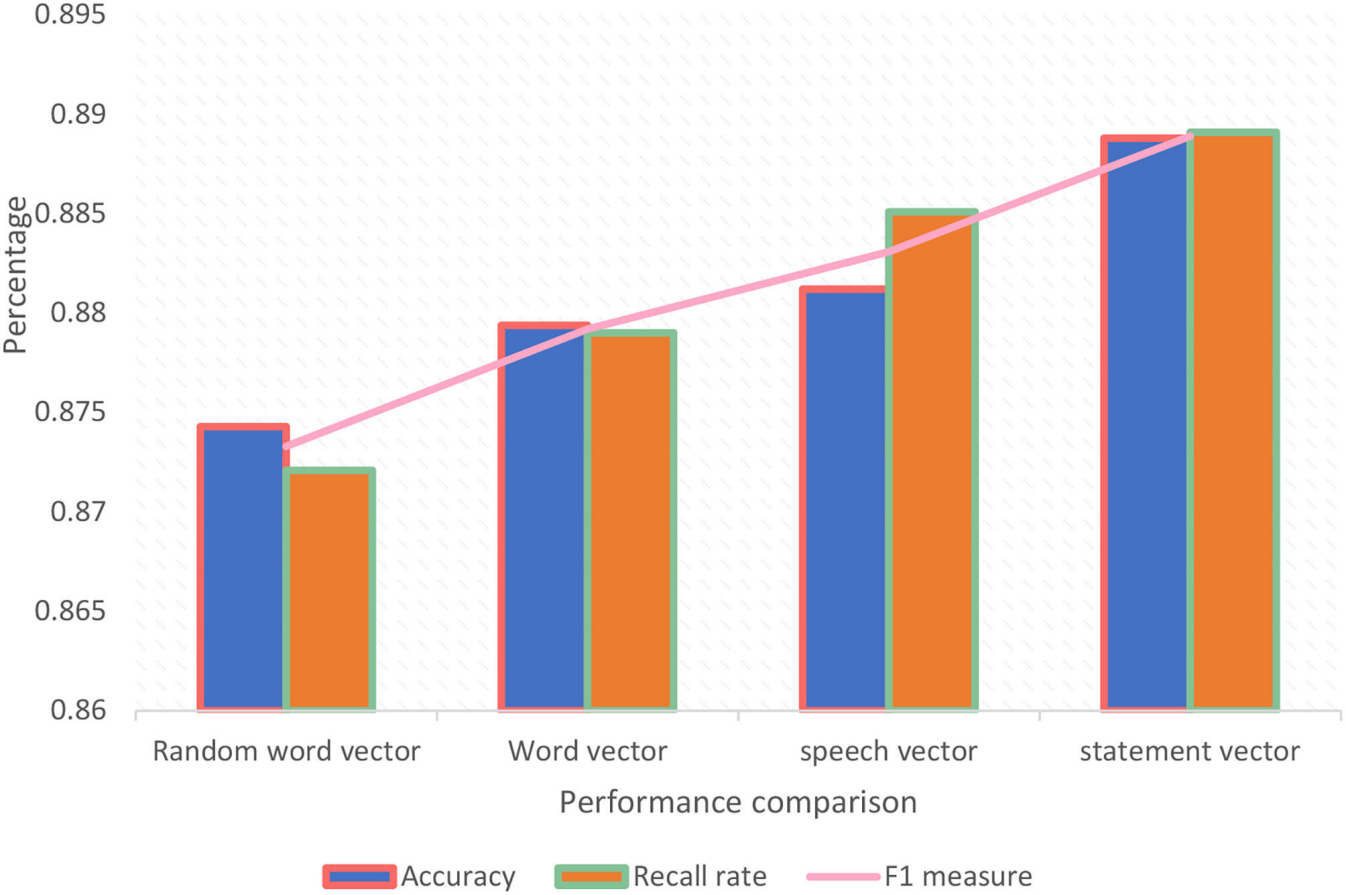
Performance comparison between models.

As shown in Figure 6, it is found that the result of using word vector training is better than the training result of a random word vector. The reason is that in the training process, the context information between the words can be considered to get a better representation, thereby improving the text sentiment analysis, the accuracy, and other effects of the comparison; the training result of the comparison word vector is better than the training result of the word vector. The reason is that in the Chinese language, compared to the word, the word is a more effective collection of semantic information. This training method can be more effective. It effectively represents the semantic information of the data, thereby obtaining more meaningful relevant features in the text sentence, and improving the effectiveness of text sentiment analysis; the result of word vector training is better than the result of word vector, because for Chinese, in the training process of the word vector, the words in the context are also introduced, so that in the training process, the context space of the word vector can contain the semantic information of the words in the data, which can make the semantic information of the context richer and obtain better word representation, thereby improving the effect of text sentiment analysis.

### Algorithm Analysis Based on Improved Text Sentiment Calculation

In the text-based sentiment calculation analysis, the improved naive Bayes algorithm is used. In this article, the 10-fold crossover method is selected to evaluate the performance of the classifier, and the classification accuracy results are averaged to the final classification results. The higher the average, the better the accuracy.

**Figure 7:**
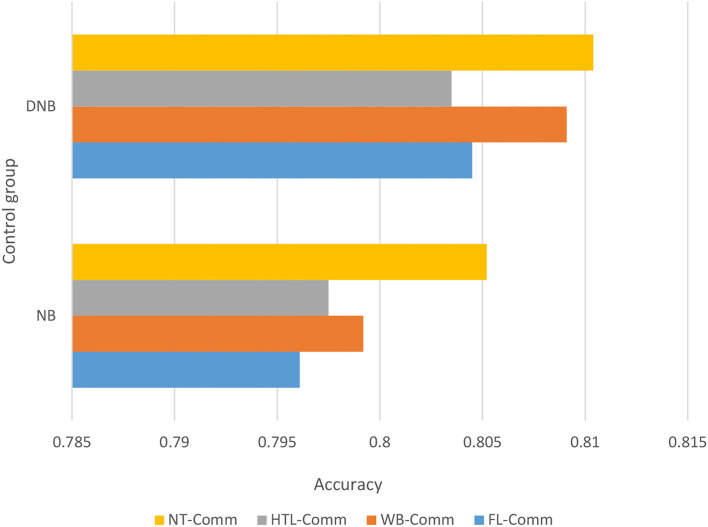
Comparison of accuracy of different classifiers on different data sets.

As shown in Figure 7, the improved naive Bayes algorithm has a higher accuracy rate than the traditional Bayes algorithm. The improved Naive Bayes algorithm, based on Euclidean distance weighting, can give different weights to the final classification results according to different attributes to improve the classification performance of the algorithm, thereby improving the classification accuracy of the algorithm. Finally, it can be concluded that the improved naive Bayes algorithm is more effective. The optimized Bayesian algorithm uses the existing data information, and the prior probability obtained by weighting the attributes replaces the traditional naive Bayes prior probability, which improves the classification accuracy. Then, transplant the improved weighted naive Bayes classifier to the Spark platform, and use caching mechanism of Spark and the operation of the resilient distributed datasets (RDD) conversion operator to greatly speed up the execution time, reflecting the advantages of parallelization of the program running in the Spark cluster.

**Figure 8:**
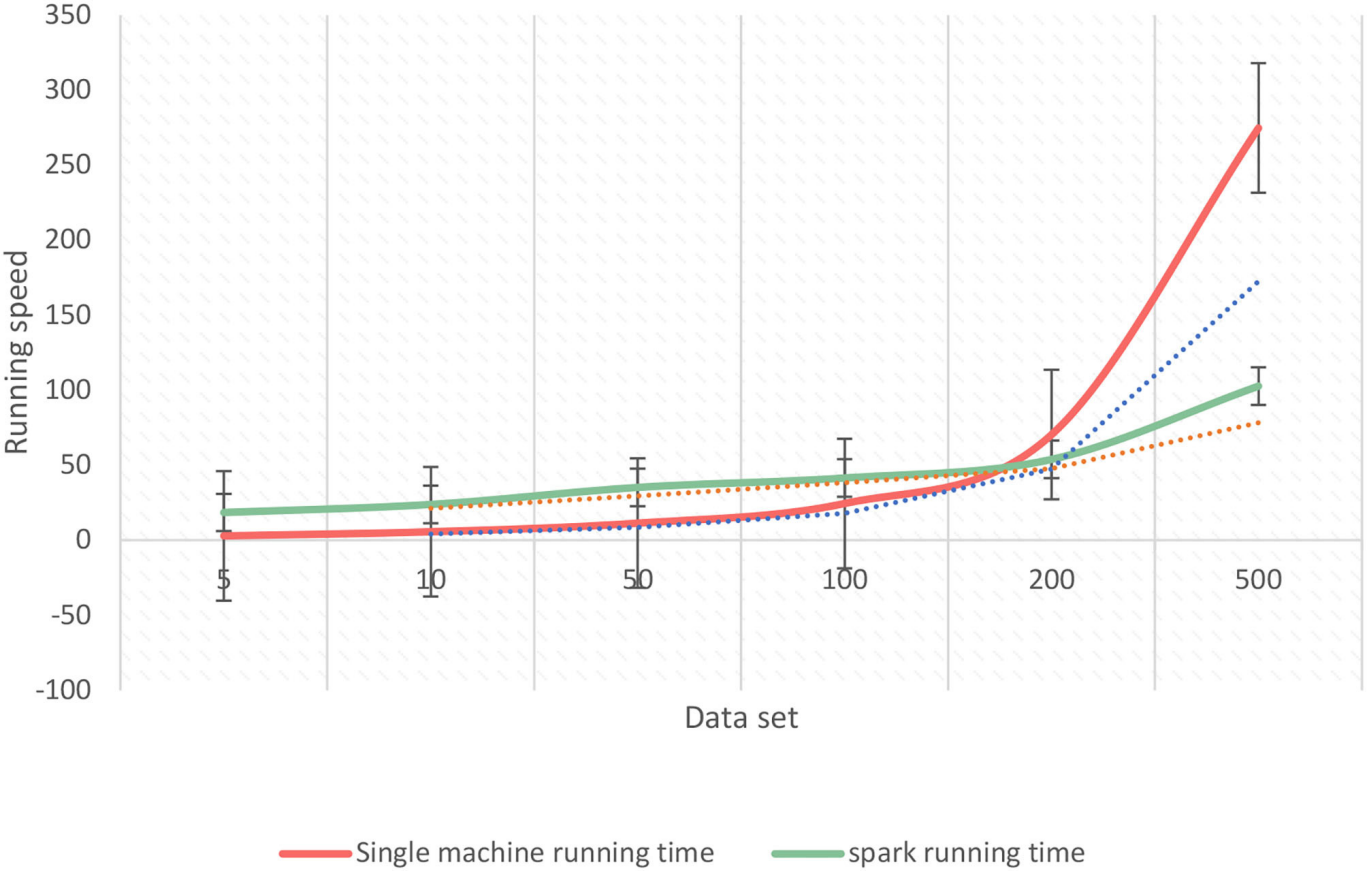
Comparison of running time of different scale data sets.

As shown in Figure 8, under different data scales, the running speed of a single machine and the running speed under the Spark platform have their own merits. On a scale of <100,000, the running speed of a single machine is faster, because, between nodes under the Spark platform, communication will consume time and will consume more time in communication and scheduling. On the scale of more than 100,000, the running speed of the Spark platform will be faster than that of a single machine, because the Spark platform will divide the data set into many small pieces, then divide it into different tasks according to different types, and then distribute according to the principle of data locality. Therefore, the traditional algorithm will affect the classification ability of Naive Bayes on the assumption of independence, and the optimized Naive Bayes improves the classification accuracy.

As shown in Table 3, Spark has achieved better results in the selected theme emotional trend sequence. The Spark model + native model proposed in this article is slightly lower than the Spark model but much higher than the native model, which illustrates this article proposes the validity of the model.

**Table 3:** Comparison of root mean square error and model training time result comparison.

**Model**	**RMSE**
Single machine running time	517.381
Spark running time	173.805
Single machine + Spark running time	209.712

**Figure 9:**
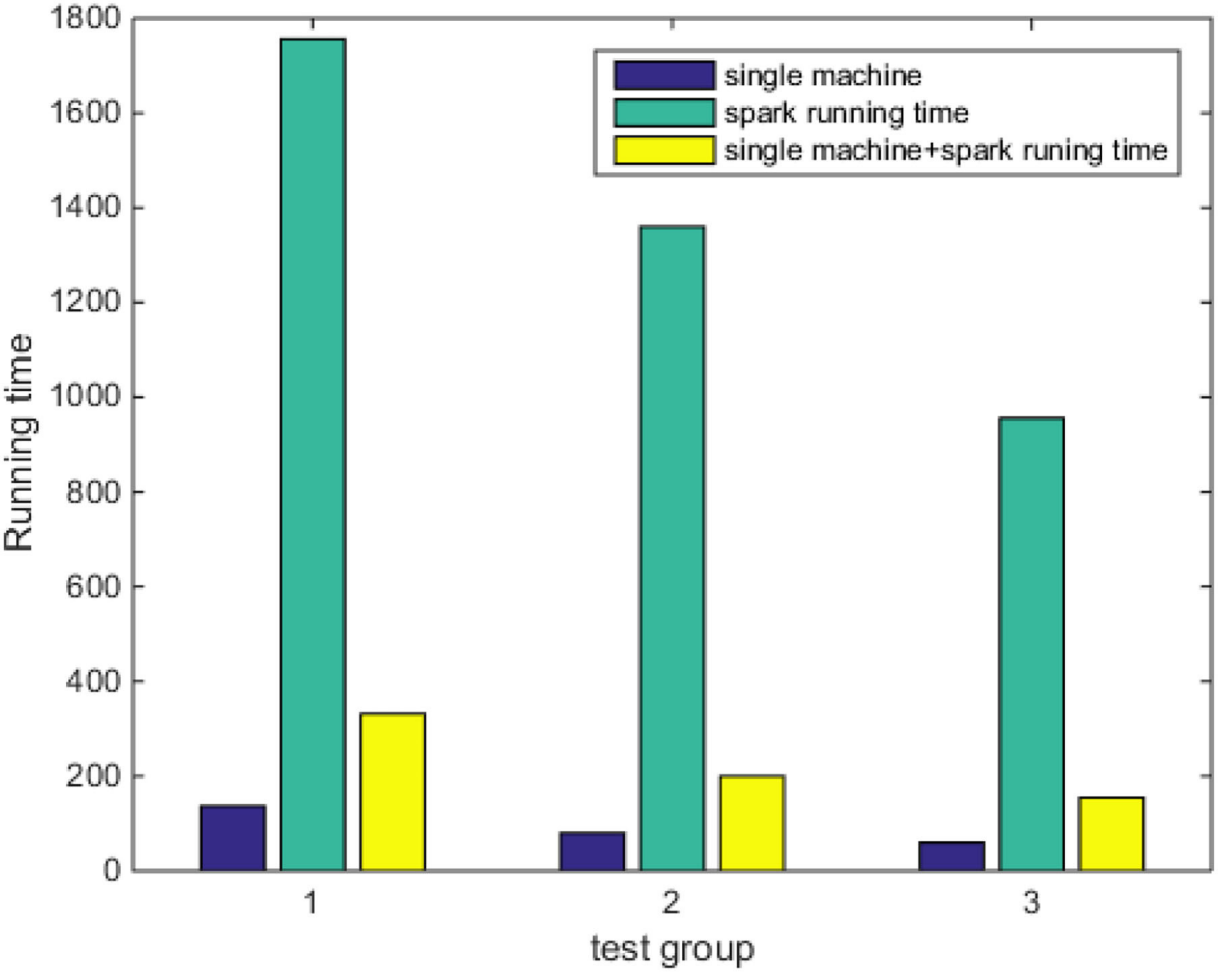
Comparison of model training time.

As shown in Figure 9, when comparing three sets of experiments, the training time of the Spark model is much lower than the other two models, and the training time of the native model is the longest. In summary, although the Spark model loses part of its accuracy, it greatly shortens the model training time and is more in line with the scenario of public opinion trend prediction. When the data set is increased to a certain extent, memory overflow and other problems will occur in a stand-alone environment, and Spark will greatly accelerate the execution speed because of the caching mechanism and the operation of the RDD conversion operator, which reflects the advantages of parallelization of the program running in the Spark cluster. Therefore, the experiment shows that the classifier based on the Spark platform has an advantage in the running speed of the algorithm over the classifier on a stand-alone machine.

## Conclusions

With the abundance of Internet resources and the vigorous development of mobile Internet. People's participation in the Internet is increasing. Due to the active participation of people, the digital entrepreneurial ecosystem has great development potential, but because these data exist in an unstructured way. Guidance lacks the ability of text sentiment analysis. Therefore, if emotional information can be extracted from these unstructured data, it will greatly promote the ability of text sentiment analysis, which has great value in scientific research and practical applications. With the rapid development of artificial intelligence technology and the maturity of large-scale data computing frameworks, digital entrepreneurship ecosystems have also become possible. This article mainly studies the ability of text emotional word extraction and trend prediction technology based on the digital entrepreneurial ecosystem and models and analyzes and predicts text data. The optimized Bayesian algorithm is used, which has a higher accuracy rate than the traditional Bayesian algorithm. Using the optimized Bi-LSTM model, the accuracy, recall, and F1 values have been slightly improved. On the Spark platform running down, the training time is greatly reduced. In the digital entrepreneurial ecosystem designed in this article, many functions are not perfect due to the short time. In the next step, it is anticipated to improve the functions of the system.

## Data Availability Statement

The original contributions presented in the study are included in the article/supplementary material, further inquiries can be directed to the corresponding author/s.

## Author Contributions

JL: writing–editing. MY: data analysis. Both authors contributed to the article and approved the submitted version.

## Conflict of Interest

The authors declare that the research was conducted in the absence of any commercial or financial relationships that could be construed as a potential conflict of interest.

## Publisher's Note

All claims expressed in this article are solely those of the authors and do not necessarily represent those of their affiliated organizations, or those of the publisher, the editors and the reviewers. Any product that may be evaluated in this article, or claim that may be made by its manufacturer, is not guaranteed or endorsed by the publisher.
